# Surgeon-Performed Endoscopic Retrograde Cholangiopancreatography in a Rural Trinidad Hospital: A 10-Year Review of Practice and Outcomes

**DOI:** 10.7759/cureus.114206

**Published:** 2026-08-09

**Authors:** Thane D Guerra, Krystan Hosein, Sarai Koon Koon, Ryan Richardson, Alan De Freitas, Dale Hassranah

**Affiliations:** 1 Department of General Surgery, Sangre Grande Hospital Campus, Sangre Grande, TTO

**Keywords:** biliary diseases, endoscopic retrograde cholangiopancreatography, hepato-pancreato-biliary surgery, post-endoscopic retrograde cholangiopancreatography pancreatitis, post-ercp complications, therapeutic endoscopy

## Abstract

Background: Endoscopic retrograde cholangiopancreatography (ERCP) is a key therapeutic modality for biliary and pancreatic diseases. While outcomes from tertiary centres are well documented, there is limited published data describing ERCP practice in Caribbean settings. This study evaluated the patient characteristics, indications, procedural outcomes, and complications of surgeon-performed ERCPs at a rural hospital in Trinidad.

Methods: A retrospective observational study was conducted of all patients who underwent attempted or completed ERCP between 1 January 2015 and 31 December 2025, performed by general surgeons at a rural hospital in eastern Trinidad. Patients with incomplete records or abandoned procedures before cannulation were excluded. Demographic characteristics, indications, procedural findings, therapeutic interventions, technical success, procedure failures, and 30-day complications were extracted from operative records and medical files. Continuous variables were summarised using medians with interquartile ranges (IQRs), while categorical variables were reported as frequencies and percentages.

Results: Seventy-nine ERCPs performed in 75 patients were analysed. Median age was 54.0 (IQR: 36-67) years, consisting of 52 females (69.3%) and 23 males (30.7%). Choledocholithiasis was the predominant indication. Deep biliary cannulation was achieved in 83.5% of procedures. Stones were identified in 54 patients and successfully extracted in 51 of those (94.4%). Therapeutic intervention was performed in 84.8% of procedures, with stone extraction (64.6%) and sphincterotomy (58.2%) being the most common interventions. Thirteen procedures (16.5%) were unsuccessful, primarily due to failed cannulation, inadequate sedation, or intra-operative desaturation. Thirty-day complications occurred in 21.5% of procedures, the most common being mild post-ERCP pancreatitis (15.2%). Bleeding occurred in 3.8%, cardiopulmonary complications in 2.5%, and cholangitis in 1.3%. No perforations or procedure-related mortality was identified.

Conclusion: Surgeon-performed ERCP in a rural Caribbean hospital is safe and effective in this specific rural setting when performed by trained surgeons with appropriate support, with technical success and complication rates approaching British Society of Gastroenterology benchmarks, though below the European Society of Gastrointestinal Endoscopy's 90% target. The predominance of therapeutic procedures, high stone clearance rate, and the absence of procedure-related mortality suggest feasibility of decentralisation of advanced endoscopic services in a resource-limited Caribbean setting, pending further multi-centre validation. These findings provide one of the first benchmarks for surgeon-performed ERCP outcomes in the English-speaking Caribbean.

## Introduction

Endoscopic retrograde cholangiopancreatography (ERCP) is an advanced endoscopic procedure that uses fluoroscopy to diagnose and treat disease within the biliopancreatic system [1]. The first published use of ERCP in the Caribbean was in 2004 by Plummer et al. at the University Hospital of the West Indies, Jamaica [2]. Over time, ERCP has evolved from a primarily diagnostic tool into a predominantly therapeutic procedure with advances in training and techniques improving safety and clinical outcomes.

Successful ERCP requires deep cannulation of the common bile duct and/or main pancreatic duct via the ampulla of Vater and the completion of the necessary therapeutic intervention [3]. Therapeutic use of ERCP is indicated in the management of choledocholithiasis, pancreatic duct stones, benign and malignant stricture of the biliary tree, bile and pancreatic leaks, sphincter of Oddi dysfunction, and stent placement and associated complications [4]. ERCP allows the endoscopist to employ several interventions to achieve diagnostic and therapeutic success. These include biliary or pancreatic sphincterotomy, stone removal, stricture dilatation, tissue sampling, and biliary and/or pancreatic duct stent placement [5]. Surgeon-performed and gastroenterologist-performed ERCPs are technically identical procedures, with differences primarily reflecting each speciality's training and clinical focus rather than the procedure itself. Surgeons often integrate ERCP with operative management of pancreaticobiliary disease, whereas gastroenterologists focus on advanced therapeutic endoscopy; however, outcomes are generally comparable when procedures are performed by experienced, high-volume operators [6].

Despite modern advances, ERCP is technically complex with a relatively high complication rate. Systematic reviews show complication rates as high as 15.9% and a mortality rate ranging between 0.26% and 1.0% [7]. Cardiopulmonary events, bleeding, infection, acute pancreatitis, and perforation are the more commonly noted complications associated with ERCP [8]. Post-ERCP pancreatitis (PEP) is the most common complication seen with an incidence rate of 4%-14% [6,9].

Most ERCP outcome data originate from high-volume tertiary centres in developed healthcare systems and are commonly performed by gastroenterologists. In contrast, there is limited published data describing ERCP practice in Caribbean settings, particularly those performed by surgeons. Furthermore, ERCP is not readily available in the Caribbean. A report to the Caribbean College of Surgeons in 2023 stated that only four countries in the English-speaking Caribbean had ERCP readily available [10]. Evaluating ERCP practice, complication rates, and outcomes in rural hospitals is critical for monitoring and optimising procedural safety and informing service development. This study aimed to (1) describe the patient demographics and indications for ERCP at a rural Trinidad hospital; (2) evaluate technical success rates and therapeutic interventions performed; (3) assess 30-day complication rates; and (4) compare outcomes with established international benchmarks to determine the feasibility of surgeon-performed ERCP in a resource-limited Caribbean setting.

This paper was presented as an oral presentation on 3 July 2026 at the Caribbean College of Surgeons 24th Annual Scientific Conference.

## Materials and methods

Study design

To address the study objective of evaluating the indications, outcomes, and complications of surgeon-performed ERCP at a rural Caribbean hospital, we conducted a 10-year, retrospective, observational, descriptive study. All ERCPs were performed or supervised by a board-approved surgical specialist.

Study setting

This study was performed at a public hospital in Sangre Grande, Trinidad. The procedures were performed in the operating theatre with portable fluoroscopy and supported by the dedicated endoscopy unit consisting of nurses, a radiographer, and a physician anaesthesiologist.

Study duration

Data were collected for all ERCP procedures performed between 1 January 2015 and 31 December 2024. Data abstraction and analysis were undertaken from April to June 2026 following institutional ethical approval.

Study population

The study population comprised all patients who underwent an attempted or complete ERPC during the study period. Patients were identified using operative theatre logbooks.

Sampling technique

A consecutive total population sampling technique was employed. All eligible ERCP procedures performed during the study period were included without randomisation or sampling.

Inclusion criteria

Patients were eligible if they underwent complete or attempted ERCP at our institution between 1 January 2015 and 31 December 2024 and complete perioperative records were available for analysis.

Exclusion criteria

Patients were excluded if ERCP was scheduled but abandoned before an attempt at cannulation of the ampulla of Vater, essential medical records or operative documentation were unavailable, or duplicate records of the same procedure were identified.

Data collection

Patient information was extracted from operative logbooks, operative notes, and medical records using a standardised data collection form. Variables collected included patient demographics (age and sex), indication for ERCP, procedural findings, therapeutic interventions performed, biliary cannulation success, stone extraction success, procedure failure and documented reasons, peri-procedural complications, and hospital outcomes. Thirty-day complication data were collected via review of hospital medical records, and no patient interviews were performed.

For the purposes of this study, Cotton et al.'s definition of post-ERCP pancreatitis was used, i.e., (1) new or worsened abdominal pain combined with (2) greater than three times the normal value of amylase or lipase at more than 24 hours after ERCP and (3) a requirement for admission or prolongation of planned admission by two to three days [11].

Statistical analysis

Data were entered into and analysed using Microsoft Excel® (Microsoft Corporation, Redmond, WA). Continuous variables were assessed for completeness and summarised using medians with interquartile ranges (IQRs). Categorical variables were summarised using frequencies and percentages.

Ethics

Exempted approval was granted by the Eastern Regional Health Authority Research Ethics Committee (ERHA-REC.013/03/2026). All identifiable patient information was removed and handled in accordance with the health authority’s guidelines.

## Results

**Figure 1:**
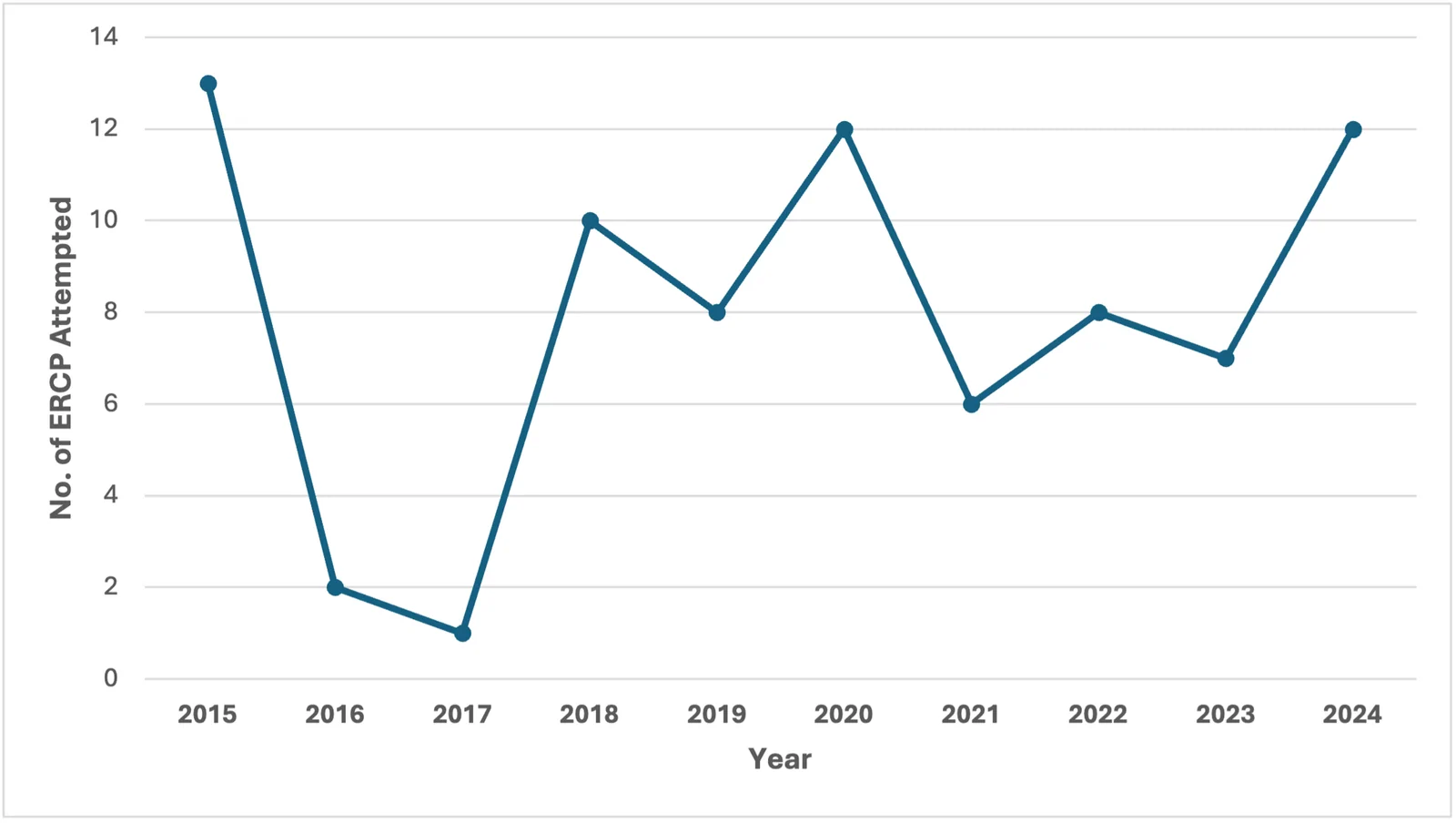
Distribution of endoscopic retrograde cholangiopancreatographies (ERCPs) performed from 2015 to 2024.

During the period from 1 January 2015 to 31 December 2024, a total of 81 consecutive patients were subjected to 85 ERCPs, all performed by surgeons. Of the 81 patients, medical records were available for 75 patients, allowing for analysis of 79 ERCPs. Figure 1 displays the number of ERCPs performed per year included in this study.

**Figure 2:**
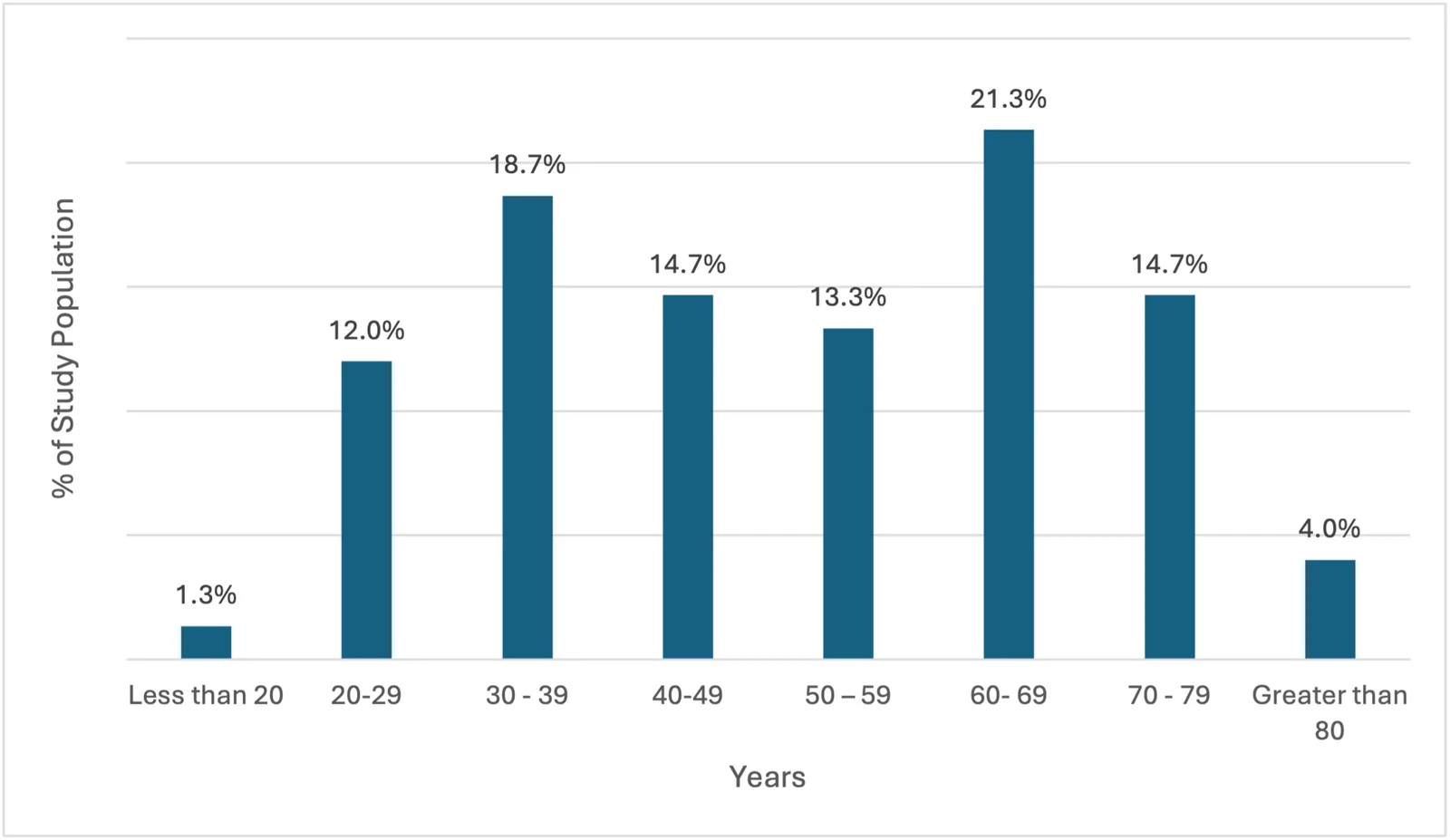
Age distribution of patients who underwent endoscopic retrograde cholangiopancreatography.

The median age of the study population was 54.0 (IQR: 36-67) years, consisting of 52 females (69.3%) and 23 males (30.7%). Figure 2 illustrates the age distribution of the 75 individuals included in the study. The majority of patients (21%) were between 60 and 69 years of age, while there was only one patient (1.3%) below the age of 20.

**Figure 3:**
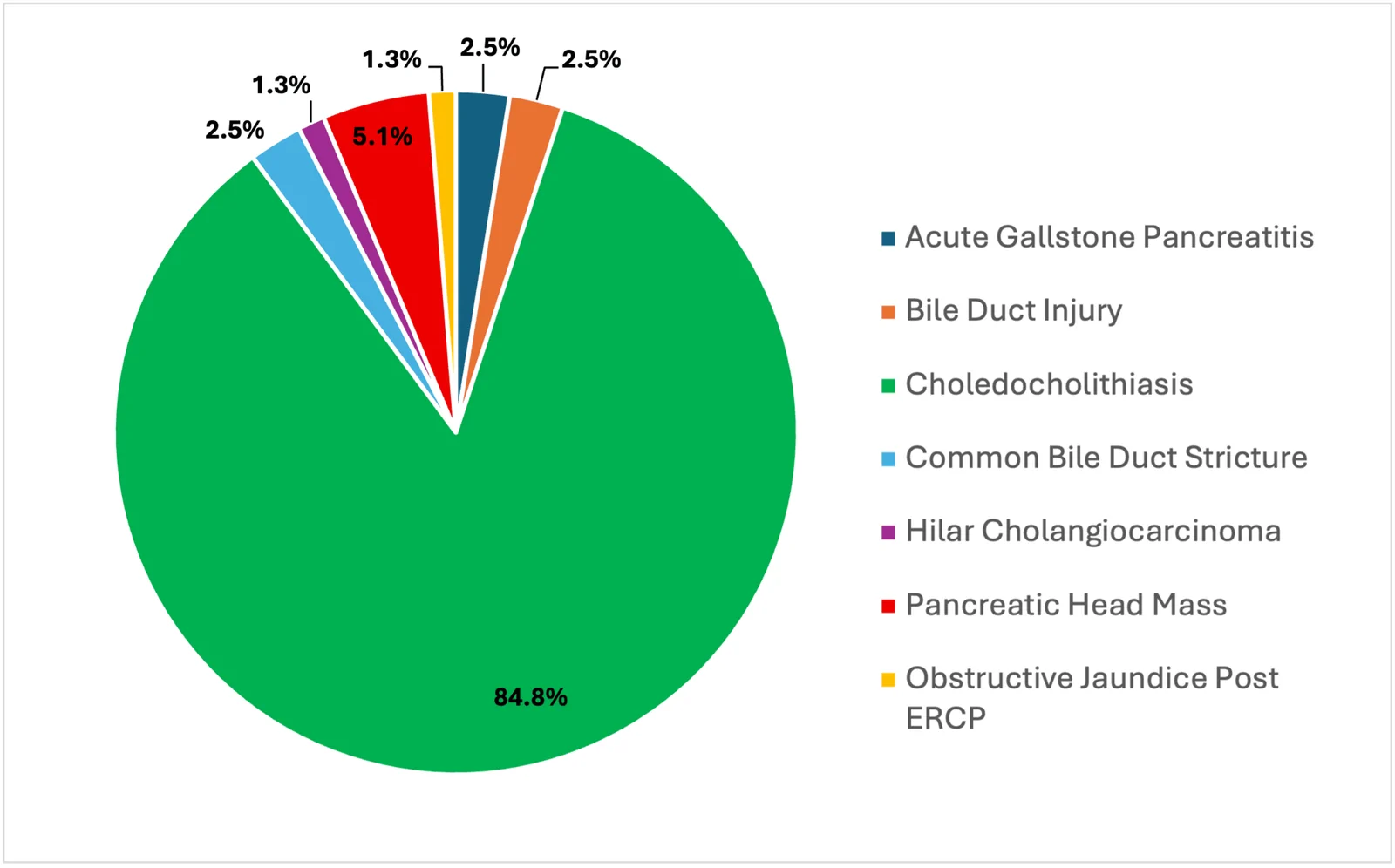
Indications for endoscopic retrograde cholangiopancreatography (ERCP).

The most common ERCP indication was suspected or confirmed choledocholithiasis in 67 (84.8%) of 79 ERCPs. Figure 3 outlines the indications for the procedure. Other indications include the management of pancreatic head masses in four (5.1%), acute gallstone pancreatitis in two (2.5%), post-ERCP obstructive jaundice in one (1.3%), hilar cholangiocarcinoma in one (1.3%), and injury and stricture of the common bile duct (CBD) in two patients each (2.5%). Notably, four patients had repeat ERCP, two had recurrence of choledocholithiasis, one had obstructive jaundice with no findings on magnetic resonance cholangiopancreatography and a history of ERCP for choledocholithiasis, and another had ERCP due to choledocholithiasis with a history of CBD stricture.

Successful cannulation of the CBD was achieved in 67 (83.5%) patients. Table 1 presents the findings during ERCP (n = 79). Of the 69 patients with suspected stone disease, biliary stones were identified in 54 patients (68.4%). Dilated CBD was seen in 14 cases (17.7%), abnormal papilla was noted in seven (8.9%), and one patient (1.3%) had a dilated intrahepatic duct. Perforations and CBD strictures were identified in two (2.5%) patients each. Purulent drainage (pus) was seen in three patients (3.8%), while five patients had biliary sludge/debris (6.3%).

**Table 1:** Findings during endoscopic retrograde cholangiopancreatography. CBD: common bile duct.

Findings	n	%
Stone on cholangiogram	54	68.4%
Male	17	21.5%
Female	37	46.8%
Abnormal papilla	7	8.9%
Biliary sludge/debris	5	6.3%
CBD stricture	2	2.5%
Dilated CBD	14	17.7%
Dilated intrahepatic duct	1	1.3%
Duodenal bulb ulcer	1	1.3%
Duodenal diverticulum	2	2.5%
Duodenal tumour	1	1.3%
Perf/leak	2	2.5%
Pus	3	3.8%
Stent in situ	1	1.3%

Therapeutic interventions were performed in 67 (84.8%) of 79 ERCPs, with some procedures having more than one intervention. Table 2 outlines the different therapeutic interventions documented in procedure notes. A minimum of one biliary stone was extracted in 51 cases (64.6%); therefore, successful stone extraction was performed for 94.4% of patients with a stone seen on cholangiogram. Sphincterotomy was documented in 46 cases (58.2%), stents were placed in six cases (7.6%), and a pre-cut papillotomy was performed in one case (1.3%).

**Table 2:** Interventions performed during endoscopic retrograde cholangiopancreatography.

Intervention	n	%
Biopsy	1	1.3%
Pre-cut papillotomy	1	1.3%
Sphincterotomy	46	58.2%
Stent placement	6	7.6%
Stone extraction	51	64.6%

Table 3 reviews the 30-day complication data of the ERCP procedures. Complications were noted in 17 (21.5%) of the 79 ERCPs performed during the study period. The most common post-ERCP complication was pancreatitis, occurring in 12 cases (15.2%). One patient was admitted for liver abscess, secondary to cholangitis, 23 days post-ERCP. Intra-operative bleeding was noted in three patients; one required intervention with diathermy; however, the other two resolved spontaneously. Two patients developed cardiopulmonary complications; however, no perforations or mortality was identified in this study population.

**Table 3:** Peri-operative complications occurring within 30 days of endoscopic retrograde cholangiopancreatography (ERCP).

Complications	n	%
Bleeding:	3	3.8%
Resolved spontaneously	2	2.5%
Resolved with diathermy	1	1.3%
Cardiopulmonary complications	2	2.5%
Cholangitis	1	1.3%
Post-ERCP pancreatitis	12	15.2%

Deep cannulation of the CBD must be achieved to classify an ERCP as successful. Based on these criteria, of the 79 ERCPs attempted/performed, 13 failed procedures (16.5%) were noted and were due to varying reasons. Table 4 reviews the reasons for failed ERCPs in this study. These include failure to cannulate the ampulla in four (5.1%) or the CBD in five (6.3%), intra-operative desaturation in two (2.6%), and inadequate sedation in two (2.6%).

**Table 4:** Reasons for endoscopic retrograde cholangiopancreatography failure.

Reason for failure	n	%
Failed to cannulate ampulla	4	5.1%
Failed to cannulate common bile duct	5	6.3%
Patient desaturation (intra-operative)	2	2.5%
Inadequate sedation	2	2.5%

## Discussion

This study evaluates ERCPs performed on Caribbean nationals, a patient population rarely analysed and documented in the literature. In this setting, choledocholithiasis was the leading indication for ERCP. This aligns with global ERCP practice patterns, including tertiary centres, where stone disease is the most common indication for intervention in the United States, United Kingdom, and Southern Asia [12-14].

The overall 30-day complication rate in this cohort was 21.5%, which includes rates of pancreatitis (15.2%), bleeding (3.8%), cardiopulmonary complications (2.5%), infection (1.3%), perforation (0%), or mortality (0%). Despite the overall complication rate appearing higher than reported in published studies, an individual comparison provides a more detailed context. The infection and bleeding rates observed in this study are comparable to or below those of previously published data, which report post-ERCP cholangitis rates of 0.5%-3% and ERCP-associated bleeding rates of 0.3%-9.6%. The rate of cardiopulmonary complications in our cohort was also lower than previously reported estimates. Literature states that these are often associated with procedural sedation and account for 4% to 16% of ERCP-related complications. The absence of perforation and procedure-related mortality in our cohort is consistent with the global low incidence of these adverse events [7,8,15].

Patients were diagnosed with PEP if they had new or worsened abdominal pain combined with greater than three times the normal value of amylase or lipase at more than 24 hours after ERCP and a requirement for admission or prolongation of planned admission by two to three days, as defined by Cotton et al. [11]. A systematic review assessing PEP, cited by the American Society of Gastrointestinal Endoscopy (ASGE) Standards of Practice Committee, identified the incidence of PEP to be 13% in North America, with an increased incidence of 14.7% in high-risk patients [8,16]. Our study shows a PEP rate of 15.2%; however, all were mild cases with resolution within 48 hours, and no patients were noted to have systemic involvement or complications of pancreatitis.

The technical success rate of 83.5% in this study is comparable to the guidelines outlined by the British Society of Gastroenterology (BSG), which promotes a cannulation rate of 85% or greater [17]. However, there is room for improvement before achieving the target biliary cannulation rate of 90%, as recommended by the European Society of Gastrointestinal Endoscopy [18]. This reaffirms that endoscopist experience and adherence to procedural standards are the primary factors for technical success as determined by Syrén et al. [19]. The absence of perforation and procedure-related mortality in this study further supports the safety and technical quality of ERCP when performed by experienced operators even in a rural, secondary-level hospital environment.

Beyond procedural outcomes, this study has important implications for healthcare equity in Trinidad and Tobago and other small island developing states. Geographic disparities in access to subspecialty services can contribute to delayed intervention and increased morbidity. Establishing and maintaining ERCP capability in rural hospitals reduces the need for inter-hospital transfers, minimises delays in care, and alleviates demand on tertiary institutions. In developing countries and resource-constrained systems, decentralised access to advanced services will enhance overall system resilience and patient access when supported by appropriate training and quality control.

Most of the available literature arises from North America, Europe, and East Asia, where patient demographics, disease prevalence, and healthcare infrastructure differ considerably from those in the Caribbean and South America. This compilation of multi-year, locally generated data from consecutive ERCP cases contributes to the limited body of data on ERCP outcomes, establishes an important benchmark for future audits in the nation and Caribbean region, and provides a foundation for quality improvement throughout. Additionally, this is the first study to evaluate surgeon-performed ERCP in the English-speaking Caribbean. Future studies would enable more in-depth analysis of long-term outcomes, complication risk, and indicators for technical success.

Limitations

Several limitations warrant consideration. The sample size (79 procedures) is modest, which limits the ability to detect rare adverse events such as perforation. Operator experience and case volume were not systematically analysed, and the absence of patient co-morbidity data precludes risk-adjusted complication analysis. The retrospective nature of data abstraction introduces the potential for recording errors, and inter-observer reliability was not assessed. The 7.4% exclusion rate due to missing records may introduce selection bias; this is unlikely to have substantially altered the primary findings. The study’s single-centre design may limit generalisability to other institutions with different operator experience or case volume. Additionally, long-term outcomes such as stent patency or malignancy survival were not assessed. Notwithstanding these limitations, the study’s strengths include the consecutive inclusion of all eligible cases over a decade, the high therapeutic success rate (94.4% stone clearance) once cannulation was achieved, and the demonstration that complex endoscopic services can be safely delivered in a rural, resource-limited setting by trained surgeons.

## Conclusions

ERCP at our institution was predominantly therapeutic, with stone-related biliary disease being the predominant indication. High technical success in benign biliary disease, acceptable complication rates, and no procedure-related mortality suggest the feasibility of decentralisation of advanced endoscopic services in a resource-limited Caribbean setting and provide an evidence base for ongoing service development and quality improvement.
